# Quantification of muscle fiber malformations using edge detection to investigate chronic muscle pressure ulcers

**DOI:** 10.3389/fbinf.2024.1450146

**Published:** 2024-10-21

**Authors:** Charlene Z. L. Ong, N. Jannah M. Nasir, Roy E. Welsch, Lisa Tucker-Kellogg, Jagath C. Rajapakse

**Affiliations:** ^1^ Health Informatics Lab, College of Computing and Data Science, Nanyang Technological University, Singapore, Singapore; ^2^ Centre for Computational Biology and Programme in Cancer and Stem Cell Biology, Duke-NUS Medical School, Singapore, Singapore; ^3^ Sloan School of Management Operations Research and Statistics, Massachusetts Institute of Technology, Cambridge, MA, United States

**Keywords:** edge detection, deep learning, imaging biomarkers, tissue morphology, muscle fibers, pressure injuries

## Abstract

**Background:**

Microscopy of regenerated tissue shows different morphologies between the healing of acute wounds and chronic wounds. This difference can be seen manually by biologists, but computational methods are needed to automate the characterization of morphology and regenerative quality in regenerated muscle tissue.

**Results:**

From the detected edge segments, we computed several imaging biomarkers of interest, such as median tortuosity, number of edge segments normalized by area, median edge segment distance and interquartile range of orientation angles of edge segments of the microscope images of successful and unsuccessful muscle regeneration. We observed that muscle fibers in saline-treated pressure ulcers had a larger interquartile range of orientation angles of the edge segments (p = 0.05) and shorter edge segment distances (p = 0.003) compared to those of acute cardiotoxin injuries.

**Conclusion:**

Our edge detection method was able to identify statistically significant differences in some of the imaging biomarkers between saline-treated pressure ulcers and cardiotoxin injuries, suggesting that chronic pressure ulcers have increased muscle fiber malformations compared to cardiotoxin injuries.

## 1 Introduction

Tissue regeneration is responsible for repairing the function and maintaining the quality of organs. The quality of tissue regeneration depends not only on cell proliferation but on multi-cellular geometry and meso-scale morphology (Anderson, 2022), which can be assessed from microscope imaging. Computational methods are required for automated analysis of tissue regeneration, but the goals of analysis depend on the specific tissue being studied.

Muscle tissue has cylindrical fibers (myofibers) organized as bundles of straight lines. While minor damage within the cell membrane of muscle fibers is patched by fusing intracellular vesicles with the damaged sarcolemma (Kaczmarek et al., 2021), more severe muscle damage can be repaired by a myogenic process where the muscle satellite stem cells proliferate and differentiate into muscle progenitor cells. The progenitor cells then migrate toward sites of injury and fuse to create a multi-nucleated myotube. Finally, myotubes undergo a maturation process that includes lateral fusion as well as intracellular rearrangements to form myofibers (Grand and Rudnicki, 2007). When the myogenic sequence is successful, the end result is the restoration of normal (straight and parallel) myofiber morphology and contractile function. Unsuccessful muscle regeneration occurs in many genetic disorders (e.g., muscular dystrophy) and our recent work characterized abnormal muscle regeneration in chronic wounds from pressure (Nasir et al., 2022; Nasir et al., 2023).

Muscle pressure ulcers (mPU) are categorized as chronic wounds because healing is often slow or incomplete (Mervis and Phillips, 2019; Preston et al., 2017). Our recent work (Nasir et al., 2023) collected samples of injured and regenerated muscle while varying the type of injury and the type of post-injury treatment, under controlled conditions *in vivo*, namely, deferoxamine (DFO)-treated pressure ulcers, saline-treated pressure ulcers and cardiotoxin injuries. This is a comparison between acute injury (cardiotoxin), with myofibers being restored to their initial morphology after a period of time, and chronic muscle pressure ulcer, where myofibers regenerate incompletely and poorly with branched fibers even after a prolonged period of time. These recapitulate real world chronic muscle injuries where split fibers are considered as defective regeneration (Eriksson et al., 2006) and are especially vulnerable to reinjury (Pichavant et al., 2015). To provide better samples for developing precision analysis methods, confounding effects from infection and microbial colonization were prevented by conducting the entire experiment in a specific-pathogen-free facility. The resulting microscope images of successful and unsuccessful muscle regeneration provide the starting point for our current problem statement, which is to develop an automated score of regeneration quality via computational quantification of microscopic muscle morphology.

There is no current approach to automate quantification of regenerative quality based on myofiber morphology in multi-channel confocal images. Moreover, there is no formal classification of fiber malformations such as tortuosity, non-parallel structures, or the presence of split fibers (smaller or greater than 90°). In particular, tortuosity has been investigated as a biomarker in many areas, such as in the field of ophthalmology, where retinal vascular tortuosity has been studied as a biomarker for retinal and cardiovascular diseases (Poirier et al., 2024). Vessel tortuosity has also been investigated in tumor vasculature and was able to predict immune checkpoint inhibitor response (Alilou et al., 2022). These muscle fiber malformations usually appear with increased waviness or non-parallel bundles of fibers. Manual judgment of muscle fiber malformations can also be subjective, varying by scientist, and the scientific community requires a portable, invariant approach that different scientists can use. In particular, one of the main features of these muscle fibers were the edges, where straight and parallel lines were indicative of normal myofiber morphology and wavy, non-parallel and split lines were indicative of poor regenerative quality. As one of the basic features in the edge, the image edges appear to be extremely relevant in identifying these malformations. To capture the extent of split, wavy and non-parallel lines, the density of edge segments, edge segment distance, orientation of edge segments, and waviness will be of relevance in a quantitative approach.

Early methods of edge detection, such as Canny edge detection or Robert operator, often involved the use of intensity and color gradients (Canny, 1986). The Robert operator (Roberts, 1965) works as a local difference operator to detect the image contour. However, these algorithms are usually sensitive to noise and edges could be lost when excluding the noise (Rong et al., 2014). In addition, the Canny edge detector works optimally in scenarios where high-contrast edges were present, hence failing to detect edges where there was only a gradual change in brightness (Martin et al., 2004).

Due to inadequacies in methods using intensity and color gradient changes, more complex methods based on posterior probability of a boundary using features such as brightness, color and texture (Martin et al., 2004) were developed. Konishi et al. (2003) utilized presegmented images to learn the probability distributions of filter responses. Based on the filter responses, a likelihood ratio test can be conducted to detect the edges. In addition, structured learning was utilized by Dollár and Zitnick (2013) to leverage on local edge patterns such as straight lines or T-junctions to predict a structured segmentation mask. However, these approaches often utilized handcrafted features for training models to predict edges. These features might lack high level representation to capture semantically meaningful edges. In multichannel confocal images of muscle fibers, there could be low image contrast between neighbouring muscle fibers, hence rendering methods based on intensity or color gradient changes ineffective. Due to the low image contrast, there could be a presence of false negatives, in which semantically meaningful edges would be required.

Recently, the feasibility of using deep learning in Computer Vision meant that many low-level computer vision tasks, such as edge detection, could now achieve SOTA performance, with an optimal dataset scale metric reaching 0.894 on benchmark datasets such as the Multicue Dataset for Boundary Detection (Soria et al., 2021). This is coupled by the availability of datasets for developing edge detection methods, such as the Berkeley Segmentation Dataset 500 (BSDS500) dataset (Arbelaez et al., 2010). This led to an explosion of new deep-learning edge detectors using convolutional neural networks (CNN). Convolutional neural networks are a type of neural network which operate primarily on images. It comprises convolutional layers which consist of convolutional kernels which are optimized (O’shea and Nash, 2015). One of the first applications of Deep Learning was DeepEdge by Bertasius et al. (2015) via a multi-scale deep network to classify the presence of a contour and to predict the fraction of labelers in consensus to the presence of a contour at a certain point. Xie and Tu (2015) proposed the Holistically-Nested Edge Detection and resolved the detection of edges via multiple scales. Other convolutional methods include Richer Convolutional Features (RCF) (Liu et al., 2017), Deep Crisp Boundaries (Wang et al., 2018) and DexiNed (Poma et al., 2020). In particular, RCF aimed to leverage on features from all convolutional layers in CNN-based models, tapping upon the rich feature representations learned by these convolutional layers across multiple scales. RCF had been shown to outperform other deep learning and non-deep learning approaches in Optimal Dataset Scale and Optimal Image Scale metrics for the BSDS500 dataset (Liu et al., 2017). Given the SOTA performance demonstrated by RCF and to capture the rich convolutional features in different layers, we hence proposed a combination of a deep-learning based edge detection approach based on RCF together with image processing methods to derive quantitative measures of these muscle fiber malformations.

Here, we first utilized an edge detector, RCF, proposed by Liu et al. (2017) to condense all the convolutional features at different stages of their neural network. The edge detector is applied to the multi-channel confocal images of mouse muscle with cardiotoxin injury, mPU treated with saline or mPU treated with deferoxamine, to extract an edge probability map of these muscle fibers. Thereafter, post-processing is done and relevant biomarkers, such as tortuosity, number of edge segments normalized by area, are quantified to provide a quantitative measure for muscle fiber malformations.

## 2 Method

### 2.1 Mice

Animal experiments were approved by the institutional animal care and use committee of SingHealth, Singapore (SHS/2016/1,257). Five-month-old C57BL/6 mice were used for the experiments, both male and female, with n = 6 mice per group. To conditionally label the Pax7 expressing muscle satellite (stem) cells, we crossed the Pax7-Cre-ERT2 with the R26R-Confetti mouse. Once the Cre-ERT2 was activated, it would recombine the confetti construct, resulting in the random expression of one of four fluorescent proteins, mCerulean (CFPmem), hrGFP II (GFPnuc), mYFP (YFPcyt) and tdimer2 (12) (RFPcyt). CFPmem would be localized to the sarcolemma, GFPnuc would be localized in the nucleus and YFPcyt and RFPcyt would be located in the cytoplasm.

### 2.2 Injury models

**FIGURE 1:**
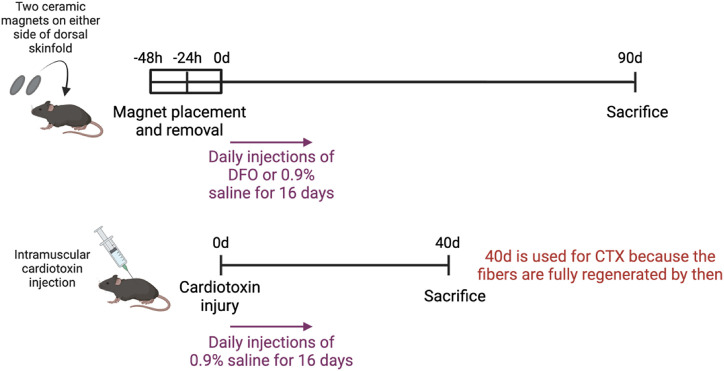
Experimental setup of various injury models.

Figure 1 illustrates the experimental setup of the various injury models. To induce muscle pressure injuries, a pair of ceramic magnets (Magnetic Source, Castle Rock, CO, part number: CD14C, grade 8) was applied to the mouse dorsal skinfold, which consists of a muscle layer, the panniculus carnosus. Pressure ulcer induction was performed in two cycles, as previously described in Nasir et al. (2023). Each cycle was made up of a 12-h period of magnet placement followed by a 12-h period without magnets. This previously established procedure induced two pressure wounds on the back of the mouse, on the left and right side of the dorsal skinfold. To treat the pressure injuries, mice were subcutaneously injected with 60 mg/kg deferoxamine (DFO) while control mice were injected with 0.9% saline for 16 days. At day 90 post-injury, mice were euthanized and wound tissues harvested, fixed in 4% paraformaldehyde (PFA), and stored until image acquisition.

At day 90 post-injury, mice were euthanized and 1 cm by 1 cm of the wounded skinfold, consisting of the skin layers and panniculus carnosus muscle, was harvested. These tissues were fixed in 4% paraformaldehyde (PFA), and stored until image acquisition. The mice which were induced with pressure ulcers and treated with DFO were referred to as the PU + DFO group, while the mice which were induced with pressure ulcers and treated with saline were referred to as the PU + saline group.

To induce acute cardiotoxin injuries, 30 μ l of 10 μ M cardiotoxin (Merck, Darmstadt, Germany) or naniproin (a cardiotoxin homologue) was injected intramuscularly into the panniculus carnosus of the dorsal skinfold of each side (left and right) of the mouse. These mice were injected with 0.9% saline for 16 days. At day 40 post-injury, mice were euthanized and 1 cm by 1 cm of the wounded skinfold, consisting of the skin layers and panniculus carnosus muscle, was harvested. These tissues were fixed in 4% paraformaldehyde (PFA), and stored until image acquisition. For cardiotoxin injured muscle, the myofibers have completely regenerated by day 40, thus day 40 was chosen as the endpoint. However, because pressure injured muscle at day 40 still showed signs of ongoing regeneration (with the presence of myoblastic cells and immature myofibers), the day 90 timepoint was chosen for the pressure ulcer group. The mice which were induced with cardiotoxin injuries were referred to as the Cardiotoxin group.

### 2.3 Image acquisition

*Ex vivo* confetti-fluorescent skin tissues, previously fixed with 4% PFA, were imaged using an Olympus FV3000 laser scanning confocal microscope (Olympus, Tokyo, Japan). Excitation and detection wavelengths used for the respective fluorophores were: CFPmem: Ex. 457 nm and Em. 466–495 nm, GFPnuc: Ex. 488 nm and Em. 498–510 nm, YFPcyt: Ex. 515 nm and Em. 521–560 nm, RFPcyt: Ex. 559 nm and Em. 590–650 nm. Each image was a 4 × 4 tile-scan taken with a 30X silicone oil immersion objective lens (NA: 1.05). Images were processed and exported using Fiji (ImageJ) software. 2-3 images were acquired from each mouse, and one representative image per mouse (making 6 images per group) was used in the downstream image analysis pipeline. The brightness and contrast of the images were adjusted using pre-set microscope settings applied to all images to account.

### 2.4 Statistical analysis

As there are three groups, a one-way ANOVA (analysis of variance) followed by the Tukey-Kramer *post hoc* test was performed. The one-way ANOVA followed by Tukey-Kramer *post hoc* test was used because the three groups are independent and the images were acquired from different animals. A comparison was made based on the biomarkers associated with the muscle fibers. Compared with multiple t-tests, an ANOVA test controls for the probability of a Type I error. In order to find out the groups which significantly differed from each other, a Tukey-Kramer *post hoc* test was performed. Tests and relevant comparison plots for biomarkers were generated by GraphPad Prism (version 9.0.0 for Windows, GraphPad Software, CA, USA). A single asterisk (*) denotes a p-value less than 0.05 (p < 0.05), double asterisks (**) denotes p < 0.01 (***) triple asterisks denotes p < 0.001 and quadruple asterisks (****) denotes p < 0.0001.

### 2.5 Manual counting by a trained biologist

A trained biologist is defined as a biologist who was trained by a board-certified veterinary pathologist to look at tissue morphology including muscle fiber malformations and has spent more than 100 h looking at brightfield (H&E) and fluorescent microscope images. The independent biologist evaluated the 18 images and counted the fiber malformations present in each image. A wavy fiber was assigned as one malformation, and one branch point in a split fiber was assigned as one malformation, where split fibers may have more than one branch point.

### 2.6 Image analysis

#### 2.6.1 Overview of our framework

**FIGURE 2:**
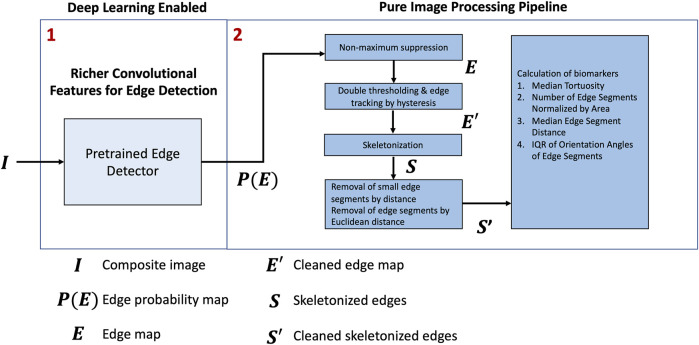
Proposed approach.

An overview of the framework is shown in Figure 2. First, we applied a pretrained model, RCF (Liu et al., 2017), to predict the edges on the composite RGB images, I , exported using Fiji (ImageJ) software. The edge probability of the composite RGB image, P(E) , was then converted into thin edges with non-maximum suppression, to obtain E . Double thresholding and edge tracking by hysteresis were performed to remove the weak edges. Next, skeletonization was done to reduce the detected edges into a 1 pixel wide representation, obtaining S . Last but not least, simple filtering based on edge segment distance and Euclidean distance was performed to obtain the final output $S^{\prime }$ , which was then used for the computation of biomarkers. In this section, the primary steps of the framework are shown below in Figure 2.

#### 2.6.2 Pre-processing

The images obtained from the FV3000 laser scanning microscope were saved in their raw formats (.oir). The files were then opened in Fiji (ImageJ). Four channels (green, cyan, yellow and red) were captured, corresponding to the confetti expression of the fibers. The brightness and contrast of the images were adjusted. A composite color image was obtained by merging the different channels and saved as a.jpg file. Due to computational constraints, the images were resized to half their original size. Similar to the pre-processing steps in RCF, the pixels were normalized in each channel by subtracting the mean intensity in each channel used in the BSDS500 dataset.

#### 2.6.3 Extraction of edge probabilities with RCF

The Richer Convolution Features for Feature Detection is adapted from the VGG16 network, where there are 13 convolutional layers and 3 fully connected layers. Liu et al. (2017) proposed that the use of rich hierarchical information could guide in the problem of edge detection. This involves the extraction of feature maps from the five stages of the convolutional network.

Let X represent the intermediate feature map after 3×3 convolution at each stage of the network, and l denote a convolutional layer of the k th stage in the network. A 1×1 convolution is performed on $X^{l,k}$ to obtain $Z^{l,k}$ .

$$Z^{l,k}=W_{x}^{l,k}X^{l,k}+b_{x}^{l,k}$$ (1)

while $W_{x}^{l,k}\in R^{F_{l}\times F_{x}\times 1\times 1}$ and $b_{x}^{l,k}$ denote the weight matrix and bias vector for layer l . The resulting feature maps $Z^{l,k}$ of the same stage are summed together. Another 1×1 convolution is performed on the summed feature maps to obtain $Z^{\prime k}$ , where $W_{z}^{k}\in R^{1\times F_{z}\times 1\times 1}$ and $b_{z}^{k}$ denote the weight matrix and bias vector for stage k performed on the summed feature maps.

$${Z^{\prime }}^{k}=W_{z}^{k}\left(Z^{l,k}+Z^{l+1,k}\right)+b_{z}^{k}$$

Thereafter, deconvolution was performed on ${Z^{\prime }}^{k}$ to obtain an upsampled feature map ${\hat{Z}}^{k}$ for k≥2 . The upsampled feature maps for each stage k were then concatenated and convolved with another 1×1 convolution matrix to obtain the fused feature maps for the 5 stages to obtain $Y^{\prime }$ . Finally, a *sigmoid* activation operation was applied to $Y^{\prime }$ to obtain the final edge probability output P(E) . The final probability output will be the same size as the given input image.

**FIGURE 3:**
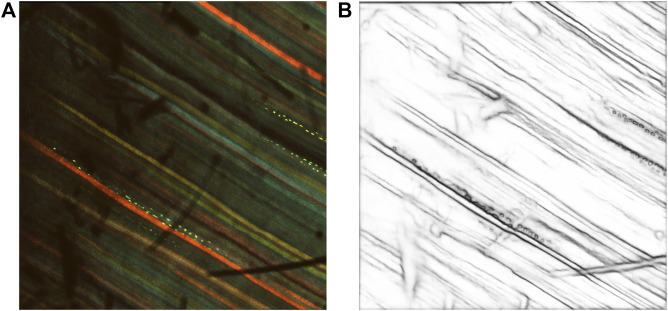
Results after prediction with RCF. **(A)** Multi-channel confocal image. **(B)** Inverse edge probability maps after prediction with RCF.

For our analysis, we refer to the implementation by the authors^1^. The pretrained model on the BSDS500 + PASCAL dataset is also provided in the Github link. We tested the pretrained model on our dataset using the multiscale edge detection configuration. Figure 3 shows the inverse edge probability map of a sample multichannel confocal image after prediction with RCF, with the original image on the left.

#### 2.6.4 Image processing of edge probability map

**FIGURE 4:**
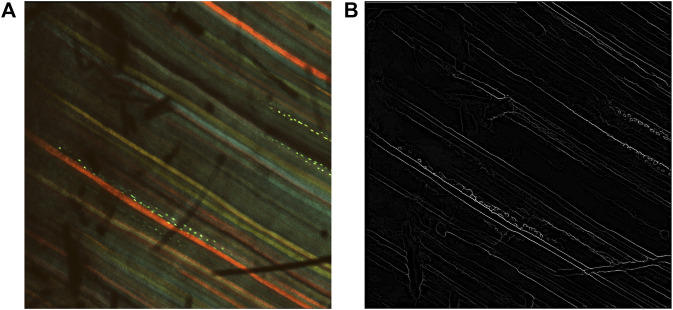
Results after non-maximum suppression. **(A)** Multi-channel confocal image. **(B)** Output after non-maximum suppression.

The inverse of the edge probability map was further processed to get the edge segments. Non-maximum suppression was implemented to the detected edges to thin the edges using Piotr’s Structured Edge Detection Toolbox (Dollár and Zitnick, 2013; Dollár and Zitnick, 2014; Zitnick and Dollár, 2014). From the non-maximum suppression, we obtained a mixture of weak and strong edges. The results obtained after non-maximal suppression can be seen in Figure 4.

In order to filter out possible noise, double thresholding was used. There are two thresholds used in double thresholding, a low threshold and a high threshold. We assign new labels to the pixels p , categorizing them as weak, strong or intermediate, according to their intensity. I(p) was compared to the low and high threshold, $t_{l}$ and $t_{h}$ . Pixels in the *strong* set are edge pixels, while pixels in the *weak* set are non-edge pixels.

$$\begin{array}{ll} p & \in \left\{strong\right\},\text{if }I\left(p\right)>t_{h}\\ p & \in \left\{weak\right\},\text{if }I\left(p\right)<t_{l}\\ p & \in \left\{intermediate\right\},\text{if }t_{l}\leq I\left(p\right)\leq t_{h} \end{array}$$

**FIGURE 5:**
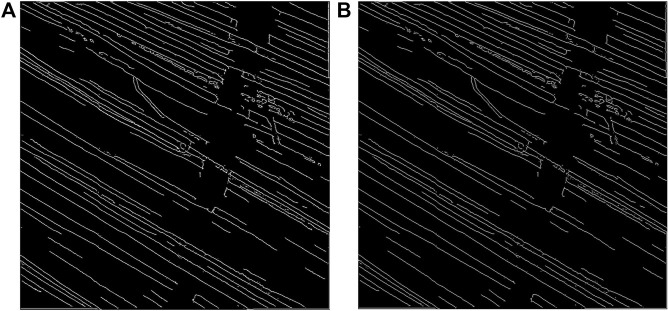
Output after **(A)** double thresholding and hysteresis tracking and **(B)** skeletonization.

Thereafter, the edges were tracked via hysteresis. This means that for any pixel in the *intermediate* set, they will be considered as an edge pixel if and only if any of their surrounding neighbors in the 8-connected neighborhood belongs to the *strong* set. Otherwise, they will be considered as non-edge pixels. The output was then skeletonized to obtain 1-pixel thick representations. Figure 5 shows the output obtained after hysteresis tracking and skeletonization.

The borders of the skeletonized output were discarded due to edge related artifacts. Next, the edge segments were characterized into different categories, namely, an endpoint-to-endpoint, junction-to-endpoint, junction-to-junction and isolated cycle, using the *skan* package (Nunez-Iglesias et al., 2018). A junction is when the edge segment intersects with another edge segment. Edge segments which are smaller than 100 microns in length and/or smaller than 50 microns in Euclidean distance are excluded.

#### 2.6.5 Quantification of biomarkers

**FIGURE 6:**
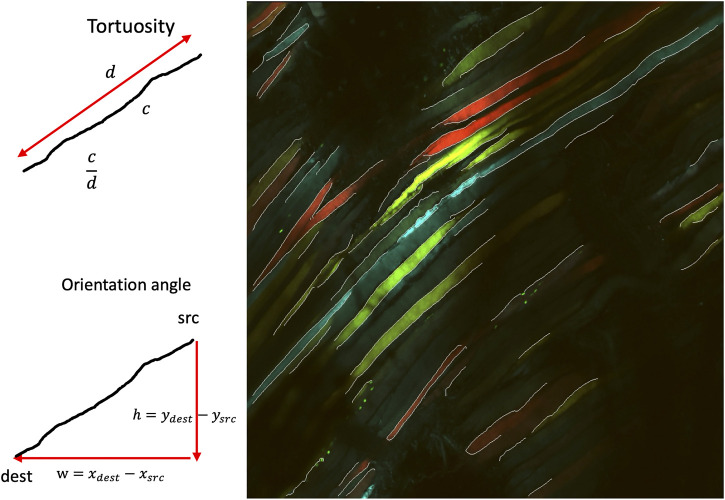
Pictorial representation of associated symbols.

The following biomarkers were quantified from the pre-processed edge segments. The biomarkers are defined as shown in table 1 [see Additional File 1]. A pictorial representation of the different associated symbols is shown in Figure 6.

**TABLE 1:** Biomarkers and their description.

Biomarkers	Description
Median tortuosity	$\frac{c}{d}$
Number of edge segments normalized by area	$\frac{N}{A}$
Edge segment distance	d
Standard deviation of orientation angles	$\sigma_{\arctan2\frac{h}{w}}$
IQR of orientation angles	$IQR_{\arctan2\frac{h}{w}}$

## 3 Results

### 3.1 Dataset

**FIGURE 7:**
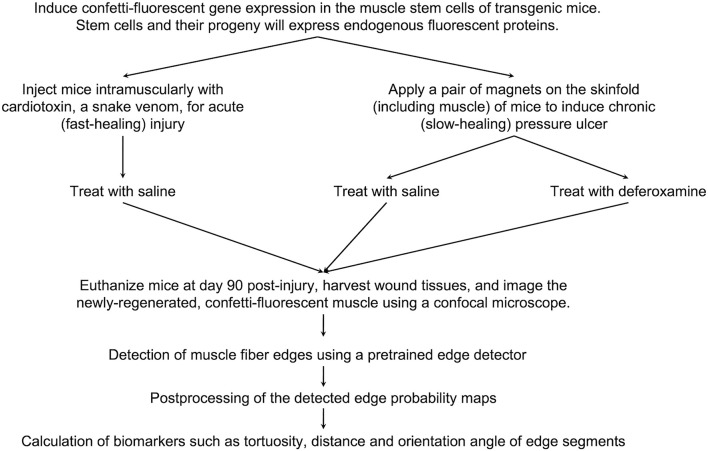
Project workflow. An overview of the murine experimental procedures, and computational methods and analysis.

The dataset used consists of multi-channel confocal images of the panniculus muscle layer of mice. For the mice with saline-treated mPU and DFO-treated mPU, they were imaged 90 days post-injury. On the other hand, mice with acute cardiotoxin injuries were imaged 40 days after injury, as regeneration was completed by that time-point. The number of images in Cardiotoxin group, PU + saline and PU + DFO groups is 6 each. As stem-cell lineage tracing is not one of the study aims, color-related information would not be relevant. Figure 7 shows an overview of the experimental and computational methods.

### 3.2 Quantification of imaging biomarkers

#### 3.2.1 Comparison of tortuosity of edge segments

The tortuosity of the edge segments is computed using the arc-chord ratio, i.e., the ratio between the length of the edge segment c and the distance between the ends of the edge segment d .

$$\tau =\frac{c}{d}$$ (2)

**FIGURE 8:**
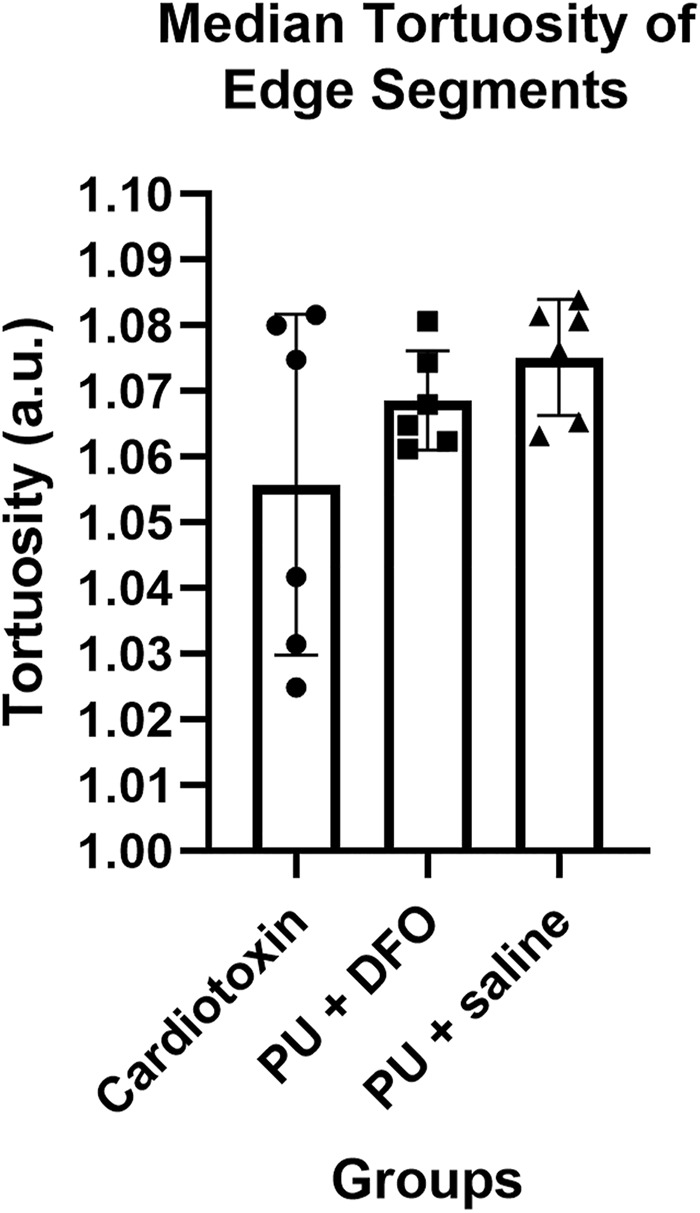
Median tortuosity of edge segments.

The median tortuosity of all the edge segments is computed for each of the images. Figure 8 compares the computed median tortuosity of all the edge segments across the three groups. We compared the median tortuosity to reduce the effects of outliers due to any misdetection caused by the shadows. It can be observed that the tortuosity of the line segments is close to 1, and hence the edge segments are close to a straight line for all the images. Due to the noise from the shadows in the Cardiotoxin images, there is no significant difference in tortuosity compared to the PU + saline group. Hence, this does not reach statistical significance.

#### 3.2.2 Comparison of number of edge segments normalized by area

**FIGURE 9:**
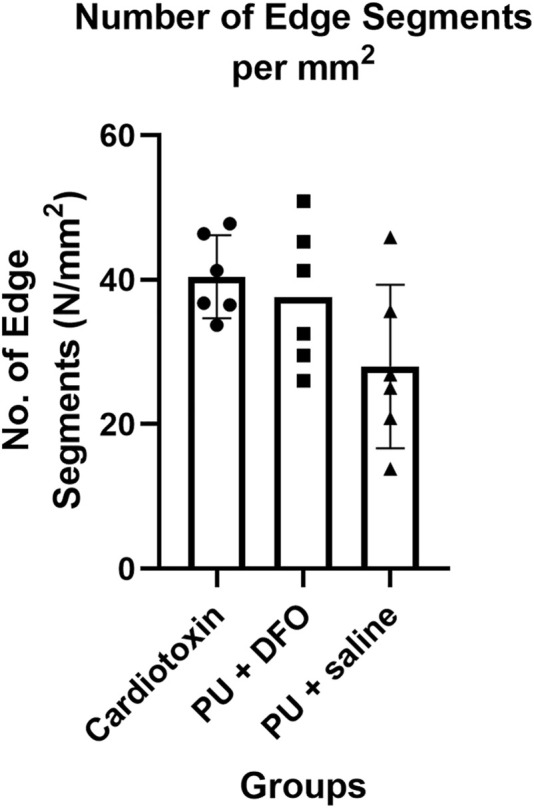
Number of edge segments normalized by area.

Another possible quantification parameter is the number of individual edge segments detected, normalized by the area of the image. Figure 9 shows the comparison of this parameter across the three groups. It can be observed that the normalized number of edge segments is lower for PU + saline compared to PU + DFO and Cardiotoxin. There is a trend between the Cardiotoxin group and the PU + saline group, with an adjusted p-value of 0.08.

#### 3.2.3 Comparison of edge segment distances

**FIGURE 10:**
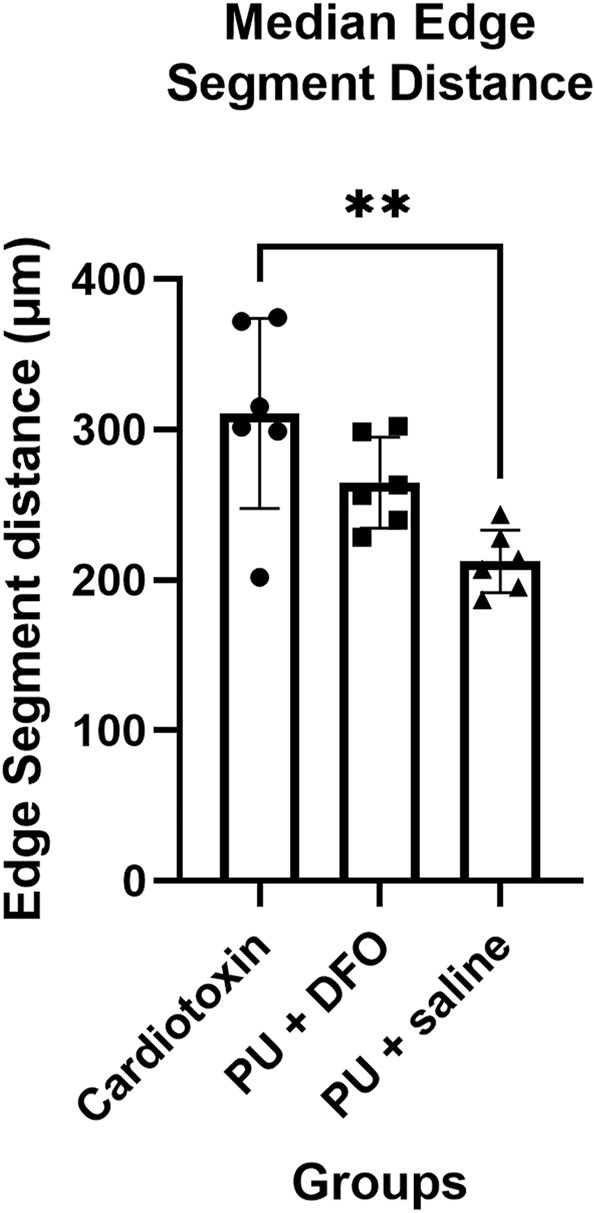
Median edge segment distance. A single asterisk (*) denotes a p-value less than 0.05 (p < 0.05), double asterisks (**) denotes p < 0.01, (***) triple asterisks denotes p < 0.001 and quadruple asterisks (****) denotes p < 0.0001.

The edge segment distance is defined as the length of the edge segment. Figure 10 shows the comparison of the edge segment distance between the 3 groups. There is a statistically significant difference between the Cardiotoxin group and the PU + saline group, with an adjusted p-value of 0.003.

#### 3.2.4 Comparison of interquartile range of orientation angles

**FIGURE 11:**
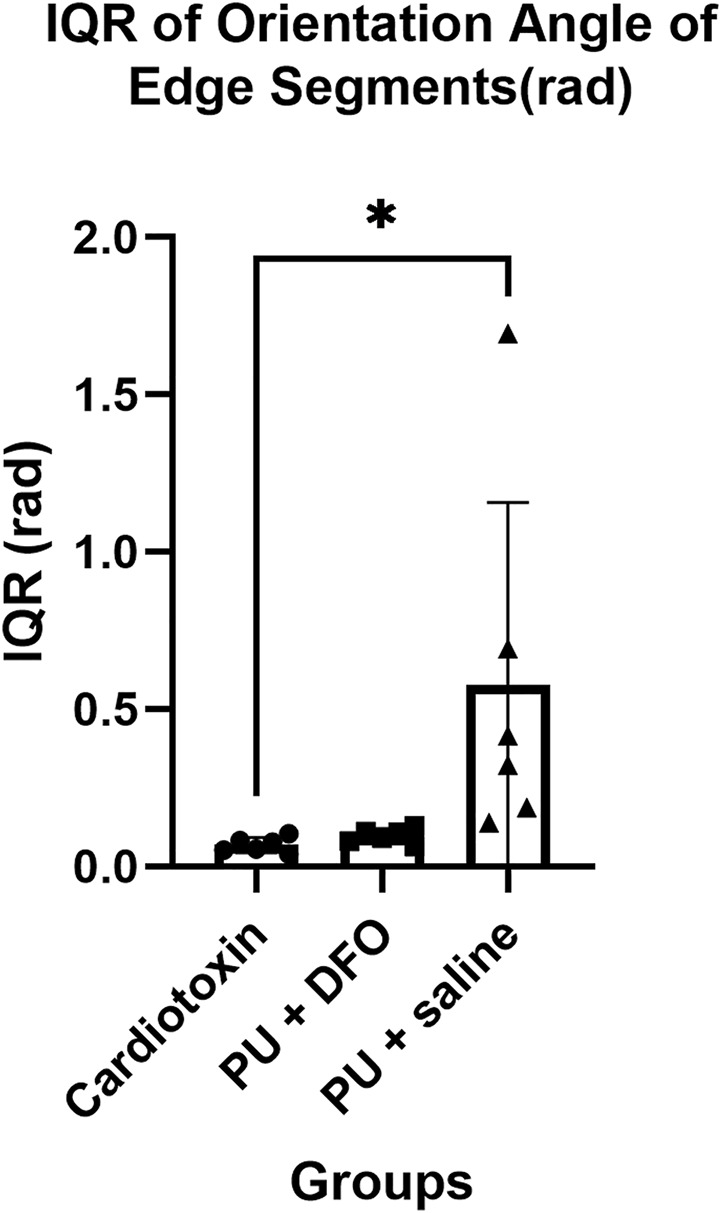
Interquartile range of orientation angles of edge segments (rad). A single asterisk (*) denotes a p-value less than 0.05 (p < 0.05), double asterisks (**) denotes p < 0.01, (***) triple asterisks denotes p < 0.001 and quadruple asterisks (****) denotes p < 0.0001.

The orientation angles of the edge segments are computed by first finding the vector that describes the edge segment orientation based on the two ends of the edge segment, followed by finding the corresponding angle of the edge segment. We postulate that normal healthy muscle fibers will be mostly parallel and hence will have a small interquartile range of their orientation angles. On the other hand, unhealthy muscle fibers will be non-parallel, bent and split, which will be a larger interquartile range of their orientation angles. Figure 11 shows the interquartile range of the orientation angles of the edge segments. We can observe that the values for PU + saline are much higher compared to PU + DFO or Cardiotoxin. When comparing between PU + saline and Cardiotoxin, there is a statistically significant difference with an adjusted p-value of 0.05. When comparing between PU + saline and PU + DFO, there is a trend with an adjusted p-value of 0.07.

#### 3.2.5 Visualizations of detected edge segments

**FIGURE 12:**
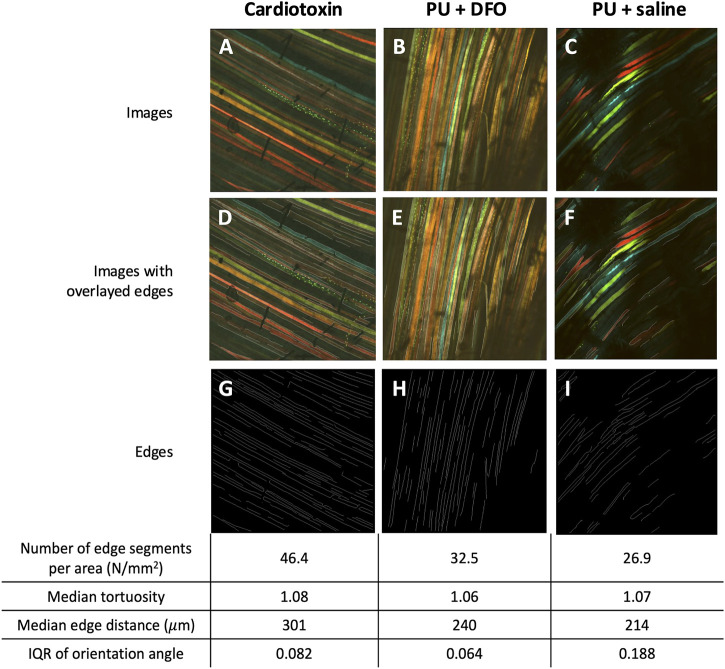
Visualizations of detected edge segments. **(A–C)** An example of multichannel confocal images of Cardiotoxin, PU + DFO and PU + saline groups respectively. **(D–F)** An example of overlaid edges on multichannel confocal images of Cardiotoxin, PU + DFO and PU + saline groups respectively. **(G–I)** An example of detected edges of Cardiotoxin, PU + DFO and PU + saline groups.

We can visualize the computed biomarkers against the original multi-channel image as shown in Figure 12 for selected images. From Figure 12, it is observed that the deep-learning enabled edge detection approach is able to pick up a number of edge segments. However, it is affected by noise associated with the shadows. For benchmarking, we also compared the results of manual counting of muscle fiber malformations by a trained biologist, against the biomarkers from our computational method in Supplementary Table S1 [see Additional File 2]. Currently, as per our knowledge, there are no available quantitative method of muscle fiber malformation. The current gold standard for quantifying the malformations was manual counting by a trained biologist. Hence, we would like to benchmark the imaging biomarkers that we have derived against the manual counting outcomes by the biologist.

## 4 Discussion

For mice induced with muscle pressure ulcers (PU), it was previously observed that the muscle failed to regenerate in the panniculus muscle layer (Nasir et al., 2022). Iron chelator, deferoxamine, or saline control were used to treat the pressure-induced injuries, and DFO-treated wounds caused improvements in long-term muscle regeneration compared to saline-treated wounds. DFO-treated wounds also displayed better muscle fiber morphology, and less frequent myofiber malformations. A high proportion of regenerated myofibers in saline-treated wound tissue was observed to be branched, split or wavy with disparate bundles of fibers, and this frequency was reduced in DFO-treated tissues. On the other hand, mice with acute cardiotoxin injuries regenerated normally and completely with straight, unbranched and parallel fibers (Nasir et al., 2023).

We were able to derive certain biomarkers that can help to quantify the differences between the three groups. Based on the expected pathological morphology of split fibers, we would expect unhealthy fibers to be oriented in different angles compared to healthy muscle fibers, which would be oriented in approximately the same direction. More parallel muscle fibers would mean a similar orientation direction, and hence a smaller interquartile range of orientation angles. Indeed, based on the results of our algorithm, we observe a statistically significant difference in the mean of the PU + saline group compared to the mean of the Cardiotoxin group for the interquartile range of the orientation angles of the edge segments. From Figure 11, it can be observed that the fibers in the PU + saline group are more split and have different orientations compared to the muscle fibers for PU + DFO or Cardiotoxin which have more fibers oriented in the same angles.

In addition, given that unhealthy myofibers are branched or split, we would expect that there would be a lower frequency of them being continuous. In healthy muscle tissue, the myotubes undergo a maturation process that includes lateral fusion and intracellular arrangement, which results in straight bundles of myofibers. When the muscle regeneration is unsuccessful, the maturation process is disrupted and the myofibers are not formed completely. In mice with cardiotoxin injuries, we would expect normal regeneration of muscle, while for mice with pressure-induced injuries, it was observed that the muscles would fail to regenerate. As a result, the edge segment distance of the PU + saline group is significantly lower than Cardiotoxin.

**FIGURE 13:**
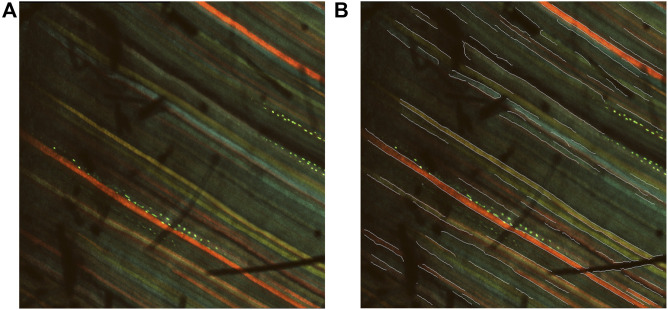
Visualizations of shadow-induced misdetections. **(A)** An example of a multichannel confocal image with shadows. **(B)** An example of a multichannel confocal image with overlaid edge segments. Shadow-induced misdetections can be observed.

We would also expect to observe a higher median tortuosity for PU + saline compared to Cardiotoxin, given increased waviness of these unhealthy muscle fibers. However, the presence of shadows caused shadow-induced misdetections in the edges, as shown in Figure 13. The number of edge segments per unit area was also expected to be lower in the PU + saline group compared to the PU + DFO or Cardiotoxin group, given worse muscle regeneration observed in the PU + saline group. Nevertheless, given that not all the edges between the muscle fibers were picked up successfully in the Cardiotoxin and PU + DFO group, there was no statistical significance observed for this biomarker.

However, albeit not statistically significant, we see a downward trend of the number of edge segments per unit area from healthy fibers (cardiotoxin injury) to unhealthy fibers (PU + saline). Similarly, we see an increasing trend of tortuosity from cardiotoxin injury to PU + saline, matching our manual analysis of myofiber deformations (Nasir et al., 2023).

In the PU + DFO group, the mean of the “number of edge segments per unit area” and mean of the “median edge segment distance” for PU + DFO was in the intermediate range of values between cardiotoxin and PU + saline, whereas the interquartile range of orientation angle of PU + DFO was comparable to the cardiotoxin group. Thus, we infer that DFO treatment of PU improved the morphology of regenerated muscle fibers and lowered the frequency of fiber malformations.

For benchmarking, we compared the results from manual counting of myofiber malformations against the computational analysis of various biomarkers in Supplementary Table S1 [see Additional File 2]. The computational method had some statistically significant comparisons and larger p values compared to manual counting by a trained biologist. A possible reason for greater p values in the computational analysis is the reduced ability of the edge detection system to identify edge segments, making visual counting by eye more effective. Manual counting showed greater statistical significance but was unable to distinguish between types of fiber malformations (e.g., tortuosity vs split fibers) and was more subjective, while the automated approach was able to provide greater granularity and enable for characterization of different type of malformations. For example, split fibers would result in an increase in interquartile range of orientation angles as these fibers would be positioned in various directions. The waviness of unhealthy fibers would be best quantified by the tortuosity of these fibers.

Nevertheless, due to the presence of shadows, edge detection might not perform most optimally and might result in the inaccurate quantification of biomarkers such as edge segment distance. To account for possible outliers due to the shadows, we have decided to compare the median edge segment distance, instead of the mean, across the three groups. Future improvements can be done to increase the robustness of the edge detection approach to the presence of shadows in the image. In addition, there is currently no validation done for the CNN model beyond comparing with the manual counting results. Further validation of our approach can be done by comparing with manual annotation of the edges, which can be used for comparison with the CNN model. Another possible limitation would be our use of Fiji for image visualization. Although Fiji provided sufficient resolution for downstream imaging biomarker analysis, its output images might pale in comparison with those from other image visualization software like Apotome 3, which we did not have access to.

In our experiments, the proposed deep-learning enabled edge-detection method was able to detect edges between the muscle fibers under different conditions, which can then be used to compute relevant imaging biomarkers that can differentiate between the Cardiotoxin, PU + DFO and PU + saline. The edges can be associated with the different muscle fibers segments and hence characterize the muscle fiber morphology. The proposed approach is the first automated approach for quantifying muscle fiber artifacts in the panniculus muscle layer, which would otherwise be counted manually and subject to between observer variability. Future improvements would include improving the edge detection approach to increase the robustness to the presence of shadows in the image.

## Data Availability

The datasets presented in this study can be found in online repositories. The names of the repository/repositories and accession number(s) can be found in the article/Supplementary Material.

## Footnotes

1 The corresponding GitHub repository is https://github.com/yun-liu/RCF-PyTorch.

## Supplementary material

**Additional File S1**
CSV file of computed biomarkers A csv file of computed biomarkers for all the samples is provided in Zenodo. It includes other biomarkers, such as the standard deviation of orientation angles of these edge segments.

**Additional File S2**
DOCX file of Supplementary Table 1 A comparison table between manual and automated counting of muscle fiber morphologies for benchmarking

## Abbreviations

mPU, muscle pressure ulcer; PU, pressure ulcer; RCF, Richer Convolutional Features; τ is defined by Equation 2; c is defined by Equation 2; d is defined by Equation 2; X is defined by Equation 1; l is defined by Equation 1; k is defined by Equation 1; Z is defined by Equation 1; W is defined by Equation 1; b is defined by Equation 1; $Y^{\prime }$ Output before sigmoid operation; I Composite image; P(E) Edge probability map; E Edge map; S Skeletonized edges; p pixels; A area; N Number of edge segments; h It is defined in Figure 6; w It is defined in Figure 6.
