# Supplementary material for: Quantification of muscle fiber malformations using edge detection to investigate chronic muscle pressure ulcers
Source: Front Bioinform. 2024 Oct 21;4:1450146. doi: 10.3389/fbinf.2024.1450146 (PMC11532102; doi:10.3389/fbinf.2024.1450146)
Supplement: Supplementary file 1 [file Table1.docx]

**Supplementary Table 1** **Comparison between manual and automated counting of muscle fiber morphologies.** Column 1 indicates the 3 comparisons performed, firstly, comparison between PU + Saline with CTX, secondly, comparison between PU + DFO with CTX and thirdly, comparison between PU + DFO with PU + saline. Column 2 shows the percentage change of myofiber malformations in the three comparison groups, CTX, PU + Saline and PU + DFO, by manual counting. Columns 3-6 show the percentage change of the different imaging biomarkers computed by our automated approach. Statistical significance was determined using a one-way ANOVA and Tukey’s posthoc test.

|  | **Manual counting by a trained biologist** | | **Automated counting by our approach** | | | | | | | |
| --- | --- | --- | --- | --- | --- | --- | --- | --- | --- | --- |
| **Comparison** | No. of malformations | | Tortuosity | | No. of edge segments per unit area | | Edge segment distance | | Interquartile range of orientation angles | |
|  | Percent change | p value | Percent change | p value | Percent change | p value | Percent change | p value | Percent change | p value |
| **PU + Saline**  **Reference Group: CTX** | Increased in PU + Saline by 930% | <0.0001 | Increased in PU + Saline by 1.8% | 0.140 | Decreased in PU + Saline by 30.7% | 0.083 | Decreased in PU + Saline by 31.6% | 0.003 | Increased in PU + Saline by 740% | 0.050 |
| **PU + DFO**  **Reference Group: CTX** | Increased in PU + DFO by 455% | 0.004 | Increased in PU + DFO  by 1.2% | 0.390 | Decreased in PU + DFO by 7.0% | 0.858 | Decreased in PU + DFO by 14.8% | 0.180 | Increased in PU + DFO by 42.3% | 0.990 |
| **PU + DFO**  **Reference Group: PU + Saline** | Decreased in PU + DFO by 46% | 0.002 | Decreased in PU + DFO by 0.6% | 0.770 | Increased in PU + DFO by 34.2% | 0.205 | Increased in PU + DFO by 24.7% | 0.110 | Decreased in PU + DFO by 83.1% | 0.070 |
