# Supplementary material for: Quantification of muscle fiber malformations using edge detection to investigate chronic muscle pressure ulcers
Source: Front Bioinform. 2024 Oct 21;4:1450146. doi: 10.3389/fbinf.2024.1450146 (PMC11532102; doi:10.3389/fbinf.2024.1450146)
Supplement: Supplementary file 2 [file Table2.docx]

**Supplementary Table 2** **List of repositories**.

| Data Type | Repository | Link / DOI |
| --- | --- | --- |
| Dataset   - Image files - Image analysis | Zenodo | 10.5281/zenodo.7071849, Version v3 |
| Code   - Richer Convolutional Features and the pretrained model for BSDS500+PASCAL | GitHub | https://github.com/yun-liu/RCF-PyTorch |
| Code   - Toolbox for non-maximum suppression | GitHub | https://github.com/pdollar/edges |
